# Endovascular Management of a Giant Skull Base Cavernous Aneurysm: Parent Artery Occlusion Is Still a Relevant Strategy

**DOI:** 10.7759/cureus.13643

**Published:** 2021-03-01

**Authors:** Mithun Sattur, Brian F Saway, Jonathan Lena, Alejandro Spiotta

**Affiliations:** 1 Department of Neurological Surgery, Medical University of South Carolina, Charleston, USA

**Keywords:** aneurysm, neurosurgery, neurosurgery training, neuroendovascular management, cavernous carotid artery aneurysm

## Abstract

Cavernous carotid aneurysms (CCAs) are usually considered benign as the natural history of the condition is often asymptomatic; however, CCAs can reach giant proportions and become symptomatic, thus requiring treatment. The introduction of flow diverters has revolutionized management of this condition. However, the parent artery geometry in giant lesions may prove exceedingly difficult to navigate and deploy stents satisfactorily. In such cases, indirect surgical treatment such as proximal occlusion of internal carotid artery (ICA) should be employed. Preoperative balloon test occlusion is indicated before permanent occlusion to identify patients who demonstrate hemispheric ischemia (for possible bypass), but it requires understanding of important operative complications and technical nuances. Endovascular parent artery sacrifice is an effective modality to achieve proximal occlusion.

Here, we describe the step-wise management approach in a 53-year-old female with a giant, left CCA presenting with headache and cavernous sinus syndrome who was ultimately successfully treated with endovascular coiling and ICA occlusion.

The management of complex lesions such as giant skull base aneurysms requires a sound understanding of vascular anatomy, tools available for evaluation, and physiological interpretation of diagnostic and therapeutic modalities to obtain excellent clinical results and patient satisfaction.

## Introduction

Giant cavernous carotid aneurysms (CCAs) are unique lesions that, when symptomatic, present with focal headaches, epistaxis, or syndromes involving cranial nerves of the cavernous sinus [1,2]. Although subarachnoid hemorrhage (SAH) is seldom a consideration, symptoms can be debilitating or potentially dangerous, thus warranting treatment. The introduction of flow-diverting stents has revolutionized the management of CCAs through endovascular means and is a form of “constructive” treatment as the internal carotid artery (ICA) is preserved [3]. Some CCAs are not amenable to this form of treatment due to technical reasons. In such cases, parent vessel sacrifice (ICA occlusion) is preferred and is currently performed by endovascular means. To determine the safety of sacrifice, a balloon test occlusion (BTO) of the ipsilateral ICA is performed beforehand [4]. In this case report, we discuss the decision-making process in a 53-year-old female with a giant CCA who underwent successful endovascular management.

## Case presentation

**Figure 1:**
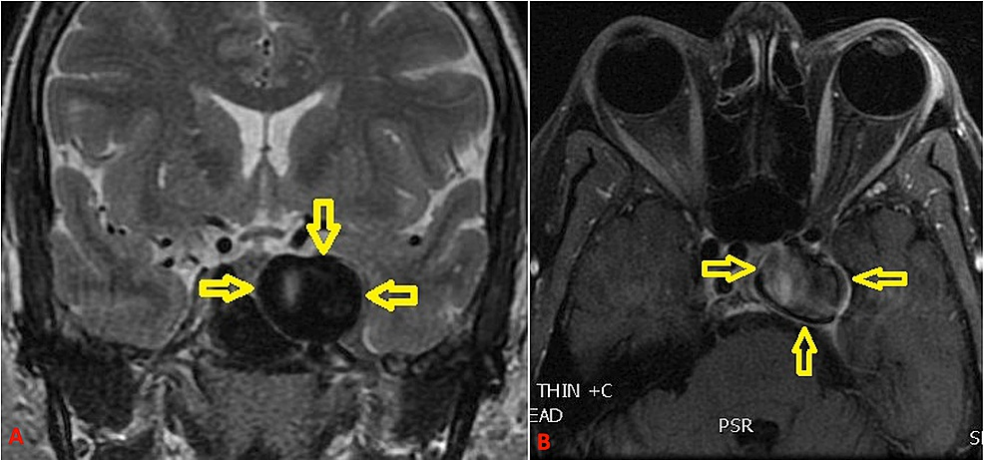
MRI obtained for clinical presentation of left cavernous sinus syndrome. Coronal T2-weighted (A) and axial T1 fat-suppressed (B) images demonstrate the lesion in the left cavernous sinus with flow void. MRI, magnetic resonance imaging

A 53-year-old female presented with subacute onset of left fronto-orbital headaches and diplopia. The patient had a medical history of hypertension and obesity (body mass index of 43.7 kg/m^2^) with no history of dyslipedemia, head or neck infections, head trauma, or radiation to the head or neck. On examination, she had partial cavernous sinus syndrome with left sixth nerve palsy and left V1, 2 hypoesthesia. Magnetic resonance imaging revealed a large lesion in the left cavernous sinus with flow voids that were highly suggestive of a giant aneurysm (Figure 1).

**Figure 2:**
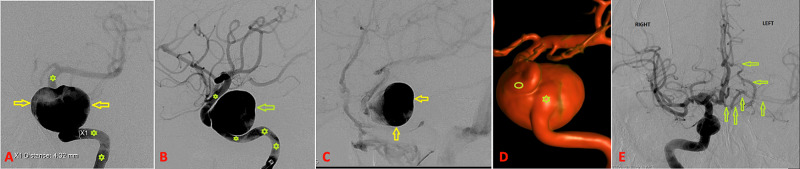
Diagnostic cerebral angiogram. AP (A) and lateral (B) left ICA angiography demonstrates the giant left CCA (arrows) with continuing profound contrast stasis in the aneurysm sac in the late venous phase (C). Asterisks indicate parent left ICA. 3D rotational angiography (D) reveals the complex geometry of the inflow zone (asterisk) and outflow zone (circle) of the aneurysm. AP view (E) obtained after right ICA injection to demonstrate collateral circulation. This shows filling of cerebral arteries (arrows) of the left hemisphere. AP, anteroposterior; ICA, internal carotid artery; CCA, cavernous carotid aneurysm; 3D, three-dimensional

Angiography was performed that revealed the aneurysm arising from the cavernous segment of the left ICA with significant stasis within the sac. Three-dimensional (3D) rotational angiography showed highly tortuous inflow and outflow channels (Figure 2).

**Figure 3:**
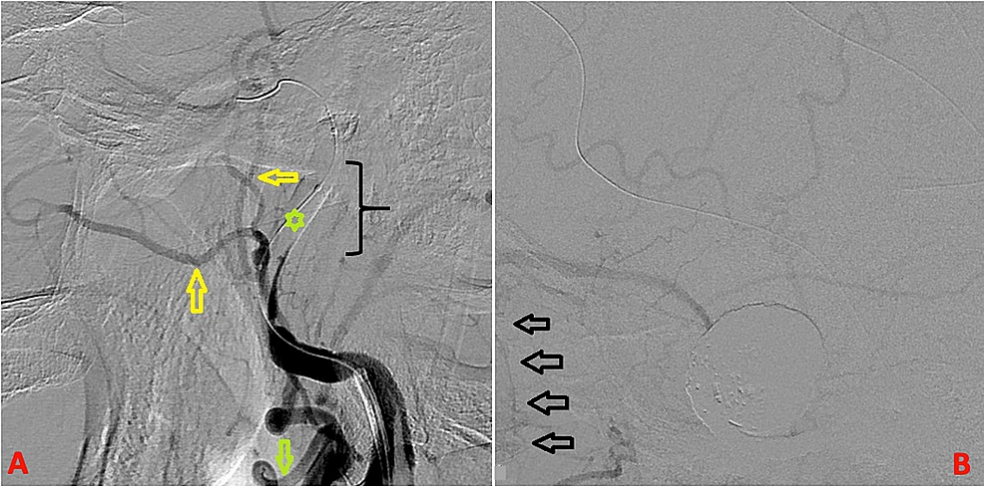
Final post occlusion images. AP X-ray (A) reveals the large coil mass (asterisk) that now occupies the giant aneurysm; arrows indicate tightly packed coils extending into the parent artery, that is, the ICA. Final angiogram from the opposite side (right ICA) is depicted in (B) and shows excellent collateral circulation to the left hemisphere through cross flow (arrow). AP, anteroposterior; ICA, internal carotid artery

Initial attempts at flow diversion with the pipeline embolization device were unsuccessful because of the parent artery geometry which made it impossible to obtain adequate and stable microcatheter purchase across the true parent artery lumen. Therefore, a decision was made to proceed with BTO for left ICA occlusion. For the BTO, she was kept awake while the balloon was navigated into the left ICA and inflated opposite the C1-2 region in standard fashion (Figure 3).

**Figure 4:**
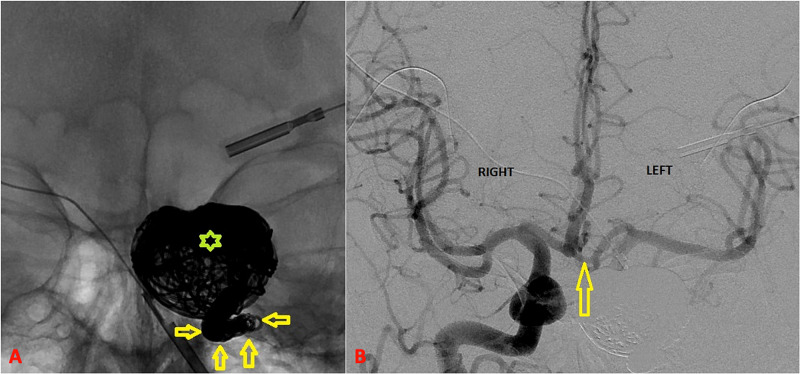
Final post-occlusion images. AP X-ray (A) reveals the large coil mass (asterisk) that now occupies the giant aneurysm; arrows indicate tightly packed coils extending into the parent artery, that is, the ICA. Final angiogram from the opposite side (right ICA) is depicted in (B) and shows excellent collateral circulation to the left hemisphere through cross flow (arrow). AP, anteroposterior; ICA, internal carotid artery

The BTO was completed without hemispheric ischemic symptoms but noted transient left eye vision change during marked induced hypotension (from mean arterial pressure 111 to 66 mmHg). Excellent left external carotid artery collaterals were observed to the left eye (Figure 3). Subsequently, under general anesthesia, the aneurysm sac was embolized with several large coils, and continuing proximally, the left proximal cavernous and distal petrous segments of the left ICA was also occluded with a vascular occlusion plug device and several smaller coils. Final angiogram revealed complete cessation of flow in the aneurysm, complete left ICA occlusion, excellent external carotid collaterals to the left eye, and excellent hemispheric cross flow (Figure 4).

She was discharged with stable preoperative deficits on aspirin 325 mg and tapering oral steroids to mitigate the mass effect-related symptoms.

## Discussion

Cavernous carotid aneurysms

CCAs are a unique subset of intracranial aneurysms that involve the cavernous section of the ICA. CCAs have been reported to account for 2-9% of intracranial aneurysms, and their etiology includes traumatic, infectious, and idiopathic [2]. Treatment of a CCA is considered when they are symptomatic and/or when they are of a large/giant size, demonstrate growth, project into the subarachnoid space, or demonstrate a bony erosion [1]. Symptoms described in the literature regarding the natural history of CCAs include cavernous sinus syndrome, carotid-cavernous fistula, epistaxis or intractable retro-orbital or fronto-orbital pain, and headache [2]. Rupture rates in large CCAs has been noted to be 9.4% at five years, but SAH is an issue on extremely rare occasions when it erodes through the dura and projects into the subarachnoid space [5]. The introduction of flow-diverting stents has revolutionized the management of CCAs by endovascular techniques. Flow diversion acts by diverting flow away from the aneurysm sac and along the parent artery, thereby inducing stasis within the aneurysm and subsequent thrombosis and ultimately endoluminal reconstruction of the parent artery across the aneurysm neck [6]. Very low mortality (0-0.44%) and morbidity rates (2.3- 3.1%) for flow diversion in CCAs have been described with the pipeline embolization device, with close to 100% occlusion rates [3]. Yet, deployment of a flow diverter may not be technically feasible due to the complex aneurysm geometry, especially in giant CCAs. Specifically, the extreme tortuosity and acute angle of the inflow and outflow channels make navigating the “true” parent artery channel without significant looping of the microcatheter within the aneurysm sac nearly impossible, which eliminates satisfactory deployment of the flow diverter. Various techniques such as stent retriever and balloon or coil anchor maneuvers have been described to overcome this but were unsuccessful in our case [7]. Thus, alternative treatment strategies need to be explored in such cases. Direct microsurgical clipping, apart from being technically extremely challenging, is associated with very high morbidity and mortality rates (14-25%) and is seldom practiced currently [3]. Endovascular coil embolization is typically successful for smaller aneurysms but is largely inadequate for giant aneurysms. A very effective strategy for giant CCAs is sacrifice of the parent artery, that is, ICA occlusion or proximal vessel ligation (Hunterian ligation). This may be performed by either microsurgical or endovascular means. The principle is flow reversal and stasis leading to aneurysm thrombosis and exclusion from circulation. Patients are subjected to a BTO procedure to assess tolerability of ICA occlusion. Studies of BTO for ICA occlusion in CCAs have shown that roughly 18-20% of the patients fail BTO and require bypass to augment hemispheric blood flow [8,9]. Endovascular proximal occlusion for CCAs has been demonstrated to be extremely effective and safe. A recent large endovascular series described 55 patients with CCAs (median size 18 mm) treated with ICA occlusion (five with bypass for failed BTO) and showed occlusion rates of 100% for 0% mortality and 2% morbidity rates, with 92% improvement in cranial nerve function [9]. The most commonly used device for ICA occlusion are coils. Detachable balloons are no longer available in the United States. Adjunctive devices such as vascular occlusion plugs may also be used in appropriate cases. In cases where a cerebral bypass is required, same-stage microsurgical proximal carotid clip occlusion, or even clip occlusion at the neck, may be achieved as an alternative to endovascular occlusion. However, the ability to coil the aneurysm sac in addition to ICA occlusion in simultaneous fashion, as in our case, makes the endovascular option attractive.

Balloon test occlusion

The sensitivity of BTO may be increased by adding supplemental techniques for assessment of cerebral blood flow (CBF) of the hemisphere, such as Xenon-computed tomography or single-photon emission computed tomography during occlusion but requires specialized equipment and protocols. Induction of hypotension is an effective method of similarly assessing CBF indirectly with the promise of avoiding false passes with the test, thus identifying patients with latent ischemia [4].

Vision and balloon test occlusion

There are certain nuances with the interpretation and significance of vision changes during BTO. Study of the external carotid collateral supply to choroidal blush of the eye during angiography is helpful [10]. Targeted ophthalmic artery (OA) occlusion as part of the BTO may be achieved by navigating and inflating the balloon opposite the OA origin intracranially, instead of at the standard high cervical (C1-2) location. This is applicable for aneurysms involving the ophthalmic segment of ICA or OA, where coiling may compromise the OA [10]. However, some advocate inflating the balloon in the ophthalmic segment for all BTOs to avoid retrograde OA filling of the ICA during balloon inflation in cases of planned sacrifice of the supraclinoid ICA intracranially above the skull base. This prevents false pass results. These were not a concern in our patient because of: (a) the transient nature of the vision change; (b) appearance during profound hypotension without hemispheric ischemic symptoms; (c) demonstration of adequate external carotid artery collaterals to choroidal blush during BTO; and (d) planned occlusion significantly below the level of OA.

**Figure 5:**
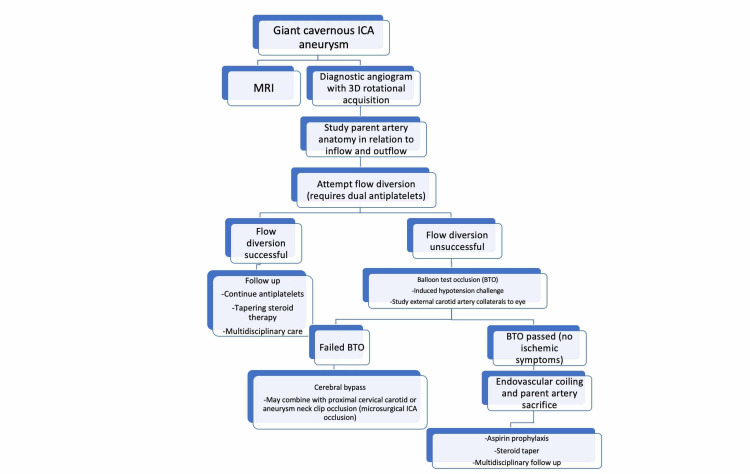
Proposed flow chart for the management of giant skull base aneurysms of the ICA. ICA, internal carotid artery

Based on our experience with giant CCAs as well as the proposed management strategies reported in the literature, we have developed a flow chart for management of giant skull base aneurysms of the ICA (Figure 5) [2,5-10].

## Conclusions

We describe the step-wise management approach in a 53-year-old female with a giant, left CCA presenting with headache and cavernous sinus syndrome who was treated with endovascular coiling and ICA occlusion and discharged home with stable preoperative deficits. As the treatment modalities for CCAs continue to evolve, it is essential that a systematic approach to treatment is defined to assure optimal clinical results. This case report provides evidence that management of giant aneurysms of the skull base segments of the ICA should be approached with a systematic algorithm beginning with a thorough diagnostic angiogram, including 3D rotational imaging to define the angio-architecture of the parent artery in relation to the aneurysm sac. Furthermore, Figure 5 provides a succinct algorithm for treatment of giant skull base aneurysms of the ICA, including CCAs. Parent artery preservation with flow diversion is currently the preferred first-line strategy, but the specific geometry of the aneurysm may render device deployment unsuccessful. In such cases, endovascular proximal parent artery sacrifice is the best treatment. This should be preceded by a high-quality BTO, incorporating the nuances of interpretation of hemispheric blood flow and vision changes. Successful outcome requires interdisciplinary management between the interventional/neuroendovascular team, neurosurgery, neuroanesthesiology, and ophthalmology, along with close clinical and imaging follow-up.
